# The eICU Collaborative Research Database, a freely available multi-center database for critical care research

**DOI:** 10.1038/sdata.2018.178

**Published:** 2018-09-11

**Authors:** Tom J. Pollard, Alistair E. W. Johnson, Jesse D. Raffa, Leo A. Celi, Roger G. Mark, Omar Badawi

**Affiliations:** 1 Institute for Medical Engineering and Science, Massachusetts Institute of Technology, Cambridge, MA 02139, USA; 2 Beth Israel Deaconess Medical Center, Boston, MA 02215, USA; 3 Department of eICU Research and Development, Philips Healthcare, Baltimore, MD 21202, USA; 4 Department of Pharmacy Practice and Science, University of Maryland, School of Pharmacy, Baltimore, MD 21201, USA

**Keywords:** Databases, Health care, Health care

## Abstract

Critical care patients are monitored closely through the course of their illness. As a result of this monitoring, large amounts of data are routinely collected for these patients. Philips Healthcare has developed a telehealth system, the eICU Program, which leverages these data to support management of critically ill patients. Here we describe the eICU Collaborative Research Database, a multi-center intensive care unit (ICU)database with high granularity data for over 200,000 admissions to ICUs monitored by eICU Programs across the United States. The database is deidentified, and includes vital sign measurements, care plan documentation, severity of illness measures, diagnosis information, treatment information, and more. Data are publicly available after registration, including completion of a training course in research with human subjects and signing of a data use agreement mandating responsible handling of the data and adhering to the principle of collaborative research. The freely available nature of the data will support a number of applications including the development of machine learning algorithms, decision support tools, and clinical research.

## Background & Summary

Intensive care units (ICUs) provide care for severely-ill patients who require invasive life-saving treatment. Critical care as a subspecialty of medicine began during a polio epidemic in which large number of patients required mechanical ventilation for many weeks^1^. Since then, the field of critical care has grown, and continues to evolve as demographics shift toward older and chronically sicker populations^2^. Patients in ICUs are monitored closely to detect physiologic changes associated with deteriorating illness that might require reassessment of the treatment regimen as appropriate. Close observation of ICU patients is facilitated by bedside monitors which continuously stream huge quantities of data, but relatively small portions of these data are archived for clinical documentation^3^. Challenges of archiving these data include integration of disparate information systems and building a comprehensive system to handle all types of data^4^.

A telehealth ICU, or teleICU, is a centralized model of care where remote providers monitor ICU patients continuously, providing both structured consultations and reactive alerts^5^. TeleICUs allow caregivers from remote locations to monitor treatments for patients, alert local providers to sudden deterioration, and supplement care plans. Philips Healthcare, a major vendor of ICU equipment and services, provides a teleICU service known as the eICU program. Care providers primarily access and document data in an information management system called eCareManager and additionally have access to the other information systems present in the hospital. After implementation of the eICU program, large amounts of data are collected and streamed for real-time monitoring by a remote ICU team. These data are archived by Philips and transformed into a research database by the eICU Research Institute (eRI)^6^.

The Laboratory for Computational Physiology (LCP) at MIT partnered with the eRI to produce the eICU Collaborative Research Database (eICU-CRD), a publicly available database sourced from the eICU Telehealth Program (Data Citation 1). The LCP has previously shared the Medical Information Mart for Intensive Care (MIMIC) database^7,8^. The latest version, MIMIC-III, contains rich deidentified data for over 60,000 ICU admissions to the Beth Israel Deaconess Medical Center in Boston, MA. MIMIC-III has been used for educational purposes, to investigate novel clinical relationships, and develop new algorithms for patient monitoring. The source hospital of MIMIC-III does not participate in the eICU program, so eICU-CRD is a completely independent set of data collected from a large number of hospitals located within the United States. The release of eICU-CRD is intended to build upon the success of MIMIC-III and expand the scope of studies possible by making data available from multiple centers.

## Methods

### Database structure and development

The eICU Collaborative Research Database is distributed as a set of comma separated value (CSV)files which can be loaded into any relational database system. Each file contains data for a single table, and we denote references to tables by using *italicized font*. Similarly, we denote references to columns using monospace font.

All tables are deidentified to meet the safe harbor provision of the US Health Insurance Portability and Accountability Act (HIPAA)^9^. These provisions include the removal of all protected health information (PHI), such as personal numbers (e.g. phone, social security), addresses, dates, and ages over 89. When creating the dataset, patients were randomly assigned a unique identifier and a lookup key was not retained. As a result the identifiers in eICU-CRD cannot be linked back to the original, identifiable data. All hospital and ICU identifiers have also been removed to protect the privacy of contributing institutions and providers.

The schema was established in collaboration with Privacert (Cambridge, MA), who certified the re-identification risk as meeting safe harbor standards (HIPAA Certification no. 1031219-2). Subsequent to this certification, free-text fields were scanned for personal information using a previously published rule-based approach^10^. Briefly, this approach scans text for known patterns indicating presence of PHI (e.g. words following ''Mr.'' are frequently names, such as ''Mr. Smith''). The approach also detects words which are commonly used as places or names. The output of this algorithm was reviewed, and rows containing potential PHI were deleted. Finally, large portions of all tables were manually reviewed by at least three personnel to verify all data had been deidentified. Frequently, due to a low number of unique entries (e.g. when a table stored the results of a drop-down menu), the entire table was reviewed.

The schema of eICU-CRD is highly denormalized. All tables can be accessed independently and linked to a single patient tracking table, *patient*, using patientUnitStayId. The only exception to this is the hospital table, which links to the patient table using hospitalId. All tables, other than *patient* and *hospital*, have a randomly generated primary key with the suffix `id' (for example, the *diagnosis* table has diagnosisId as a primary key). This column has no physical meaning, being used only to constrain uniqueness on rows and ensure integrity of the data when loading into a database system.

### Patient identifiers

**Figure 1:**
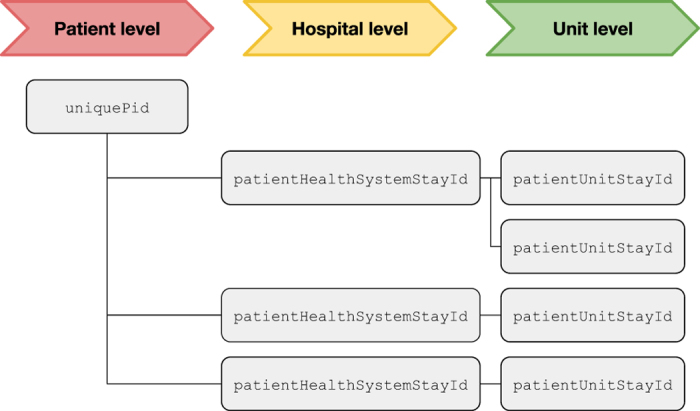
Organization of patient tracking information. Each patient is identified by a unique integer: the uniquePid. For each uniquePid, a patient may have distinct hospitalizations denoted by patientHealthSystemStayId. Finally, for each hospitalization, a patient may have distinct unit stays, denoted by patientUnitStayId. patientUnitStayId is the primary identifier used for linking data across tables.

Unit stays, where the primary unit of care is the ICU, are identified by a single integer: the patientUnitStayId. Each unique hospitalization is also assigned a unique integer, known as the patientHealthSystemStayId. Finally, patients are identified by a uniquePid. Unlike the other identifiers, uniquePid is generated using an algorithm based upon prior work on linking disparate patient medical records^11^. Each patientHealthSystemStayId has at least one or more patientUnitStayId, and each uniquePid can have multiple hospital and/or unit stays. Figure 1 visualizes this hierarchy. All tables use patientUnitStayId to identify an individual unit stay, and the patient table can be used to determine unit stays linked to the same patient and/or hospitalization.

### Sample selection

The eICU Collaborative Research Database is a subset of a research data repository maintained by eRI. A stratified random sample of patients was used to select patients for inclusion in the public dataset. The selection was done as follows: first, all hospital discharges between 2014 and 2015 were identified, and a single index stay for each unique patient was extracted. The proportion of index stays in each hospital from the eRI data repository was used to perform a stratified sample of patient index stays based upon hospital; the aim was to maintain the distribution of first ICU stays across the hospitals in the dataset. After a patient index stay was selected, all subsequent stays for that patient were also included in the dataset, regardless of the admitting hospital. A small proportion of patients only had stays in step down units or low acuity units, and these stays were removed.

### Code availability

A Jupyter Notebook containing the code used to generate the tables and descriptive statistics included in this paper is available online^12^.

The code that underpins the eICU-CRD website and documentation is openly available and contributions from the research community are encouraged^13^.

## Data Records

The database comprises 200,859 patient unit encounters for 139,367 unique patients admitted between 2014 and 2015. Patients were admitted to one of 335 units at 208 hospitals located throughout the US. Table 1 provides demographics of the dataset, including hospital level characteristics^14^.

Table 2 highlights the top 10 most frequent admission diagnoses in the dataset as coded by trained eICU clinicians using the APACHE IV diagnosis system^15^. Table 3 collapses APACHE diagnoses into 21 groups which are more clinically intuitive. Patients who are missing APACHE IV hospital mortality predictions are excluded from both tables (N=64,623). Patients will not have an APACHE IV hospital mortality prediction if they satisfy exclusion criteria for APACHE IV (burns patients, in-hospital readmissions, some transplant patients), or if their diagnosis is not documented within the first day of their ICU stay.

### Classes of data

Data include vital signs, laboratory measurements, medications, APACHE components, care plan information, admission diagnosis, patient history, time-stamped diagnoses from a structured problem list, and similarly chosen treatments. The data are organized into tables which broadly correspond to the type of data contained within the table. Table 4 gives an overview of tables available in the dataset.

### Administrative data

Hospital level information is available in the *hospital* table, and includes regional location in the USA (midwest, northeast, west, south), teaching status, and the number of hospital beds. Hospital information is the result of a survey and is sometimes incomplete: 12.5% have unknown region and 20.1% have unknown bed capacity. Table 5 shows the percentage of hospital data in each category.

Patient information is recorded in the *patient* table. The three identifiers described earlier (patientUnitStayId, patientHealthSystemStayId, uniquePid) are present in this table. Administrative information recorded in the *patient* table includes: admission and discharge time, unit type, admission source, discharge location, and patient vital status on discharge. Patient demographics are also present in the *patient* table, including age (with ages >89 grouped into `>89'), ethnicity, height, and weight.

### APACHE data

The Acute Physiology, Age, and Chronic Health Evaluation (APACHE) IV system^15^ is a tool used to risk-adjust ICU patients for ICU performance benchmarking and quality improvement analysis. The APACHE IV system, among other predictions, provides estimates of the probability that a patient dies given data from the first 24 hours. These predictions, on aggregate across many patients, can be used to benchmark hospitals and subsequently identify policies from hospitals which may be beneficial for patient outcomes. In order to make these predictions, care providers must collect a set of parameters regarding the patient: physiologic measurements, comorbid burden, treatments given, and admission diagnosis. These parameters are used in a logistic regression to predict mortality. eICU-CRD contains all parameters used in the APACHE IV equations: physiologic parameters are primarily stored in *apacheApsVar*, and other parameters are stored in *apachePredVar* . The result of the predictions for both the APACHE IV and the updated APACHE IVa equation are available in *apachePatientResult* . These data provide an informative estimate of patient severity of illness on admission to the ICU, though it should be noted that these predictions are not available for every patient, in particular: those who stay less than four hours, burns patients, certain transplant patients, and in-hospital readmissions. See the original publication for more detail^15^.

### Care plan

The care plan is a section of eCareManager which is primarily used for intraprofessional communication. The data are documented using structured multiple choice lists and the care plan is used to communicate care provider type, provider specialty, code status, prognosis, treatment status, goals of care, healthcare proxies, and end-of-life discussion.

### Care documentation

Drop down lists available in eCareManager allow for structured documentation of active problems and active treatments for a patient. It is also possible for care staff to enter short free-text entries. Eighteen tables are available in eICU-CRD which document various aspects of each patient's care including measurements made, active problems, treatments planned, and more.

#### *admissionDrug*

This table contains details of medications that a patient was taking prior to admission to the ICU. Information available includes the drug name, dosage, time frame during which the drug was administered, the user type and specialty of the clinician entering the data, and the note type where the information was entered.

#### *allergy*

Allergies were documented in the *allergy* table and sourced from patient note forms. Allergy information is available with a free text allergy name, type of documenting caregiver, whether the allergy is a drug, a standardized code for the drug (if applicable), and the time at which the allergy was documented.

#### *customLab*

Laboratory measurements that are not configured within the standard interface are included in the *customLab* table. These laboratory measurements are infrequently measured but may provide useful information for a small subset of patients. The most frequently measured test in the *customlab* table is glomerular filtration rate (GFR), and the table contains data for less than 1% of all patients in eICU-CRD v2.0.

#### *diagnosis*

Active problems were documented in the *diagnosis* table, with 86% of patients having a documented active problem during the first 24 h of their unit stay. There were a total of 3,933 unique active problems; the most common was acute respiratory failure (11.15% of patients), followed by acute renal failure (8.15% of patients)and diabetes (7.28% of patients). Problems are hierarchically categorized, and Table 6 shows the proportion of patients with an active problem for each organ system. Note that a patient can have problems documented for multiple organ systems. Most problems are mapped to International Classification of Disease (ICD) codes to facilitate identification of specific diseases using a well established ontology. However, it was not possible to map some diagnoses to ICD codes. For example, ''endocrine|glucose metabolism|diabetes mellitus|Type II|controlled'' is mapped to ICD-9 code 250.00 (Diabetes mellitus without mention of complication, type II or unspecified type, not stated as uncontrolled) and ICD-10 code E11.9 (Type 2 diabetes mellitus without complications). However, ''endocrine|glucose metabolism|diabetes mellitus'' is not mapped to an ICD code, as it is not clear whether this is type I or type II.

#### *infusionDrug*

Details of drug infusions are recorded within the *infusionDrug* table. These infusions are entered by care staff manually or interfaced from an electronic health record system from the hospital. Continuous infusions documented include vasopressors, antibiotics, anticoagulation, insulin, sedatives, analgesics, and so on. Of the 208 hospitals in eICU-CRD, 152 (73%) have data recorded in the *infusionDrug* table. Recorded information includes the name of the drug, a standardized code for the drug (using Hierarchical Ingredient Code List or HICL codes), the amount of drug in the carrying solution, the total volume of the carrier, the rate of the drug infusion, and the patient weight (if applicable for dosing). All records are stored with a single offset representing the time of the infusion.

#### *intakeOutput*

The intake and output of any volume for patients is stored in the *intakeOutput* table. Unlike the *infusionDrug* table, the aim of this table is to tabulate volume received, and thus many records exist with non-specific names such as ''Crystalloids (ml)|Continuous infusion meds''. Overall fluid balance is an important aspect of patient health, and running totals for intake, output, dialysis, and net (intake minus output)are recorded. The most frequent records in the *intakeOutput* table include urine output, infusion of normal saline, oral fluid intake, non-saline fluid administration (e.g. dextrose based), enteral feeding, parenteral feeding, and more.

#### *lab*

Laboratory values collected during routine care are interfaced with eCareManager and archived in the database. Each row of the *lab* table contains a single laboratory measurement for a patient. Each hospital has had their local laboratory measurements mapped to standard concepts. A total of 158 distinct types of laboratory measurements are captured and represented by 158 unique labName values (including ''magnesium'', ''pH'', ''BUN'', etc). Measurements are stored with the unit of measurement, the time the lab was drawn, and the last time the value was revised.

#### *medication*

Active medication orders for patients are stored in the *medication* table. When a medication order is made by a physician, a pharmacist will review and verify the order in their corresponding pharmacy system. This order verification is interfaced into eCareManager and stored in the *medication* table. Free text instructions and comments are removed during the deidentification process. In eICU-CRD, two tables focus on recording patient medication: *medication* and *infusionDrug*. There are two key differences between these tables: (1) only continuous infusions are present in *infusionDrug* (e.g. intravenously infused normal saline but not orally prescribed acetaminophen), and (2)compounds described in *medication* are orders; and while usually these orders are fulfilled and administered this cannot be guaranteed. Information available for each order includes: the start time, end time, name of the compound, HICL code, dosage, route of administration, frequency of administration, loading dose, whether the drug is given pro re nata (PRN), and whether the drug is an IV admixture.

#### *microLab*

Microbiology information from patient derived specimens is made available in the *microLab* table. Presence of bacteria in specimens such as blood or sputum provides useful information for treatment planning and selection of antibiotic regimen. For each record the time of specimen collection (e.g. blood draw), site of the culture, organism found (if any), and sensitivity to various antibiotics (if any are tested). As microbiology is documented manually by care providers, and not directly interfaced from local hospital information systems, the table is not populated for a significant number of hospitals.

#### *note*

Notes are generally entered by the physician or physician extender primarily responsible for the documentation of the patient's unit care. There are several types of notes which can be entered in the system including admission, progress, patient medical history, procedure, catheterization, and consultation. Free-text notes were removed during the deidentification process. Highly structured text notes which are selected from drop down menus are retained within the database and present in the *note* table.

#### *nurseAssessment*

The nursing assessment table stores information about the capability to assess and document patient items such as pain, psychosocial status, patient/family education, and organ specific statuses. Each record in the table is stored with the time of documentation and the time at which the assessment is relevant.

#### *nurseCare*

Patient care information is documented in the *nurseCare* table for the following categories: nutrition, activity, hygiene, wound care, line care, drain status, patient safety, alarms, isolation precautions, equipment, restraints, and other nursing care data. Each record is stored with an entry time (nurseCareEntryOffset) and a relevant time (nurseCareOffset). A custom hierarchy is used to group and store data.

#### *nurseCharting*

The majority of bedside documentation is entered into a ''flowsheet'', a tabular style interface with time in columns (usually hourly)and observations in rows. The *nurseCharting* table contains this information using a entity-attribute-value model, where the entity is a patient identifier, the attribute is the type of data recorded (e.g. heart rate), and the value is the measurement made (e.g. 80 beats per minute). Each charted item is stored with a ''chart time'' (nursingChartOffset), which specifies when the measurement was relevant, and a ''validation time'' (nursingChartEntryOffset), which indicates when the measurement was verified by staff. Vital signs available include: heart rate, heart rhythm, blood pressure, respiratory rate, peripheral oxygen saturation, temperature, location of temperature measurement, central venous pressure, oxygen flow in liters, oxygen device used for oxygen flow, and end tidal CO2. Less frequently documented vital signs available include: pulmonary artery pressure (PA), stroke volume (SV), cardiac output (CO), systemic vascular resistance (SVR), intracranial pressure (IP), cardiac index (CI), systemic vascular resistance index (SVRI), cerebral perfusion pressure (CPP), central venous oxygen saturation (SVO2), pulmonary artery occlusion pressure (PAOP), pulmonary vascular resistance (PVR), pulmonary vascular resistance index (PVRI), and intra-abdominal pressure (IAP). Other data elements available in *nurseCharting* include assessments made, commonly tabulated scores (neurological function scales, sedation scales, pain scales), and other physiologic measurements or device settings.

#### *pastHistory*

Information related a patient's relevant past medical history is stored in the *pastHistory* table. Providing a detailed past history is not common, but items such as AIDS, cirrhosis of the liver, hepatic failure, chronic renal failure, transplant, pre-existing cancers, and immunosuppression are more reliably documented due to their importance in severity of illness scoring. Elements of past medical history are documented using a custom hierarchical coding system and stored with the charted time (pastHistoryOffset) and with the entry time (pastHistoryEntryOffset).

#### *physicalExam*

Results of physical exams performed are stored in the *physicalExam* table. Data for physical exams are entered directly into eCareManager. The choices for the physical exam include "Not Performed", "Performed-Free Text", and "Performed-Structured". Free text sections are not included in the database. There is a large variety of drop-down menus for the physical exams recorded, with specific text entry boxes allowing for the creation of a structured physical exam.

#### *respiratoryCare*

This table contains information related to respiratory care. Patient data include respiratory care times, sequence of records for historical ordering, airway type/size/position, cuff pressure and various other ventilation details. Unlike other tables, the *respiratoryCare* table does not use an entity-value-attribute model, but instead has many columns for each setting, most of which are empty for a given time of data recording.

#### *respiratoryCharting*

Charted data which relate to a patient's ventilation status, including the configuration of the bedside mechanical ventilator, are stored in the *respiratoryCharting* table. Each setting is stored with an entry time (respChartEntryOffset) and an observation time (respChartOffset). Examples of settings include the percentage of oxygen inspired, tidal volumes, pressure settings, and other ventilator parameters.

#### *treatment*

A custom hierarchical coding system is used to record active treatments, and there are are 2,711 unique treatments documented in eICU-CRD. The most frequent treatments explicitly documented in the table across patients were mechanical ventilation (16.96% of patients), chest x-rays (8.79% of patients), oxygen therapy via a nasal cannula with a low fraction of oxygen (6.93% of patients), and normal saline administration (7.57%).

### Bedside monitor data

Large quantities of data are continuously recorded on ICU patients and displayed via bedside monitors. The *vitalPeriodic* and *vitalAperiodic* tables contain data derived directly from these bedside monitors. Unlike other data elements in the database, the data collected in these tables are not entered or validated by providers of care: the periodic and aperiodic vital sign data have been automatically derived and archived with no human verification.

#### *vitalPeriodic*

Continuously measured vital signs are recorded in the *vitalPeriodic* table and include heart rate, respiratory rate, oxygen saturation, temperature, invasive arterial blood pressure, pulmonary artery pressure, ST levels, and intracranial pressure (ICP). Vital signs are originally collected at 1-minute intervals, with 5-minute medians archived in eICU-CRD. Table 7 summarizes data completion for periodic vital signs. The most frequently available periodic vital sign is heart rate (available for 96% of patients), and the least available periodic vital sign is ICP (available for 0.81% of patients). Conversely, while the average number of heart rate measurements among patients with at least one recording of heart rate is 759.2 (approximately 63 hours), the average number of ICP measurements among patients with at least one ICP measurement is much higher at 1610.3 (approximately 134 hours). Thus, while monitoring of ICP is infrequent across all patients, when it is performed it results in a large number of observations.

#### *vitalAperiodic*

Aperiodic vital signs are collected at various times and include non-invasive blood pressure, pulmonary artery occlusion pressure (PAOP), cardiac output, cardiac input, systemic vascular resistance (SVR), SVR index (SVRi), pulmonary vascular resistance (PVR), and PVR index (PVRi). The most frequent aperiodic vital sign is blood pressure (available for 94% of patients), and the least frequent is PVRi (available for 0.93% of patients).

## Technical Validation

Data were verified for integrity during the data transfer process from Philips to MIT using MD5 checksums. In order to maintain data fidelity, very little post-processing has been performed. Each participant hospital in the database has customized workflows and clinical documentation processes, and as a result, the reliability and completion of data elements varies on a hospital and/or ICU level. Table 8 presents data completion across tables, showing the number of hospitals with low, medium, and high data completion.

The data archived within eICU-CRD were intended for use during routine clinical care, and not for secondary analysis. Thus, care must be taken when using the data, as inconsistencies which are inconsequential for clinical care may impact analyses performed.

A public issue tracker is used as a forum for reporting technical issues and describing solutions^13^. The correction of technical errors will be made with updated data releases.

## Usage Notes

### Data access

Data can be accessed via a PhysioNet repository^16^. Details of the data access process are available online^17^. Use of the data requires proof of completion of a course on human subjects research (e.g. from the Collaborative Institutional Training Initiative^18^). Data access also requires a data use agreement that stipulates, among other items, that the user will not share the data, will not attempt to re-identify any patients or institutions, and will release code associated with any publication using the data. Once approved, data can be directly downloaded from the eICU Collaborative Research Database project on PhysioNet.

Future updates are planned for eICU-CRD. Updates which change the schema for currently available data, and as such break code syntactically, will result in a major version change. Release of new tables, correction of issues found in currently released data, and insertion of additional data into currently available tables will result in an increment in the minor version. Due to the complexity of the deidentification process and the high sensitivity required, not all data could be made available in the current version of eICU-CRD. Updates to the current dataset will be made as data are certified safe for release. Finally, eICU-CRD v2.0 contains data for patients admitted between 2014–2015. Future updates will be made to ensure data remain contemporary.

### Collaborative code and documentation

A core aim in publicly releasing the eICU-CRD is to foster collaboration in secondary analysis of electronic health records, so we have created an openly available repository for sharing code^13^. We believe that publicly accessible code to extract reliable and consistent definitions for key clinical concepts is of utmost importance, both to accelerate research in the field and to ensure reproducibility of future studies^19,20^. Detailed documentation is available online^17^ and includes information regarding data access, table contents, and a schematic of the relationships between tables in the data. The documentation is source controlled within the code repository allowing for collaborative development^13^. Discussion around data usage, highlighting of issues, and best practices can be made via the issues panel of the GitHub repository.

### Example usage

**Figure 2:**
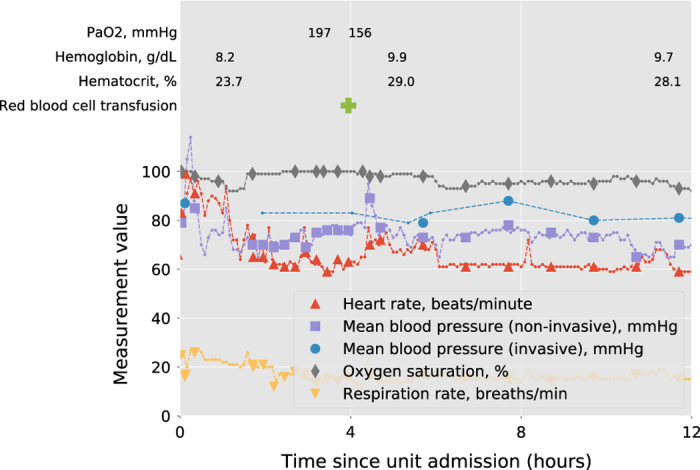
Visualization of a single patient's stay. Data shown are a subset of all data available, and include: high granularity vital signs (dashed lines, sourced from *vitalPeriodic* and *vitalAperiodic*), nurse validated vital signs (solid markers, sourced from *nurseCharting*), blood product administration (green cross, sourced from *intakeOutput*), and laboratory measurements (sourced from *lab*).

We have provided publicly accessible Jupyter Notebooks^21,22^ to demonstrate usage of the data^12^. These notebooks supplement online documentation and include a detailed review of each table, with commentary on best practices when working with the data. More general notebooks are available in the code repository referenced earlier, and include notebooks for cohort extraction, summary of demographic characteristics, and visualization of time-series data. Figure 2 visualizes of a subset of variables available during a single patient stay and can be generated using a notebook provided online^12^.

## Additional information

**How to cite this article**: Pollard, T. J. *et al*. The eICU Collaborative Research Database, a freely available multi-center database for critical care research. *Sci. Data* 5:180178 doi: 10.1038/sdata.2018.178 (2018).

**Publisher’s note**: Springer Nature remains neutral with regard to jurisdictional claims in published maps and institutional affiliations.

## Supplementary Material



## Figures and Tables

**Table 1:** Demographics of the 200,859 unit admissions in the database.

**Data**	**Median [IQR], Mean (STD), or Number (%)**
Age, years (median [IQR])	65.00 [53.00,76.00]
Unit length of stay, days (median [IQR])	1.57 [0.82,2.97]
Hospital length of stay, days (median [IQR])	5.49 [2.90,10.04]
Admission height, cm (mean (std))^a^	169.25 (13.69)
Admission weight, kg (mean (std))^a^	83.93 (27.09)
Gender (n (%))	
Male	108,379 (53.96)
Female	92,303 (45.95)
Other or Unknown	177 (0.09)
Ethnicity (n (%))	
African American	21,308 (10.61)
Asian	3,270 (1.63)
Caucasian	155,285 (77.31)
Hispanic	7,464 (3.72)
Native American	1,700 (0.85)
Other/Unknown	11,832 (5.89)
Hospital discharge year (n (%))	
2014	95,513 (47.55)
2015	105,346 (52.45)
Unit type (n (%))	
Coronary Care Unit/Cardiothoracic ICU	15,290 (7.61)
Cardiac Surgery ICU	9,625 (4.79)
Cardiothoracic ICU	6,158 (3.07)
Cardiac ICU	12,467 (6.21)
Medical ICU	17,465 (8.70)
Medical-Surgical ICU	113,222 (56.37)
Neurological ICU	14,451 (7.19)
Surgical ICU	12,181 (6.06)
Status at unit discharge (n (%))	
Alive	189,918 (94.55)
Expired	10,907 (5.43)
Unknown	34 (0.02)
Status at hospital discharge (n (%))	
Alive	181,104 (90.16)
Expired	18,004 (8.96)
Unknown	1,751 (0.87)
Note that multiple unit admissions can correspond to the same patient.	

^a^Missing data excluded from calculation.

**Table 2:** Most frequent admission diagnoses as coded using the APACHE IV diagnosis system.

**APACHE Diagnosis**	**Number of patients(%)**
Sepsis, pulmonary	6,823 (5.01)
Infarction, acute myocardial (MI)	5,919 (4.34)
CVA, cerebrovascular accident/stroke	5,284 (3.88)
CHF, congestive heart failure	4,840 (3.55)
Sepsis, renal/UTI (including bladder)	4,284 (3.14)
Diabetic ketoacidosis	4,001 (2.94)
CABG alone, coronary artery bypass grafting	3,635 (2.67)
Rhythm disturbance (atrial, supraventricular)	3,474 (2.55)
Cardiac arrest (with or without respiratory arrest)	3,377 (2.48)
Emphysema/bronchitis	3,304 (2.43)
Percentages are calculated for the subset of 136,236 unit stays with an APACHE IV hospital mortality prediction. UTI is urinary tract infection.	

**Table 3:** Most frequent categories of APACHE diagnosis using clinically meaningful groups defined in the code repository^13^.

**APACHE Diagnosis category**	**Number of patients (%)**
Sepsis	18,087 (16.40)
Cerebrovascular accident	9,758 (8.85)
Cardiac Arrest	9,135 (8.28)
Acute Coronary Syndrome	8,343 (7.57)
Respiratory medicine	7,970 (7.23)
Gastrointestinal Bleed	7,277 (6.60)
Congestive Heart Failure	5,884 (5.34)
Trauma	5,592 (5.07)
Coronary Artery Bypass Graft	4,771 (4.33)
Neurological	4,640 (4.21)
Pneumonia	4,577 (4.15)
Diabetic Ketoacidosis	4,384 (3.98)
Overdose	4,268 (3.87)
Asthma/Emphysema	3,948 (3.58)
Other cardiovascular disease	3,593 (3.26)
Valvular disorders	2,795 (2.53)
Coma	2,082 (1.89)
Acute renal failure	1,932 (1.75)
Gastrointestinal obstruction	1,232 (1.12)
Patients who are missing APACHE IV hospital mortality predictions are excluded (N=64,623, includes burns patients, in-hospital readmissions, short length of stay, and other APACHE exclusion criteria).	

**Table 4:** List of tables available in the eICU Collaborative Research Database (v2.0).

**Table name**	**Type of data**
*admissionDrug*	**Care documentation**: Medications taken prior to unit admission.
*admissionDx*	**APACHE**: Admission diagnoses and other APACHE information.
*allergy*	**Care documentation**: Known patient allergies.
*apacheApsVar*	**APACHE**: Physiology score components used in predictions.
*apachePredVar*	**APACHE**: Other components used in predictions.
*apachePatientResult*	**APACHE**: Predictions made by APACHE IV and IVa.
*carePlanCareProvider*	**Care plan**: Details regarding managing or consulting providers.
*carePlanEOL*	**Care plan**: End of life care planning.
*carePlanGeneral*	**Care plan**: Plans for patient care, often including end of life care.
*carePlanGoal*	**Care plan**: Stated goals of care for the patient.
*carePlanInfectiousDisease*	**Care plan**: Precautions for patient related to infectious disease.
*customLab*	**Care documentation**: Infrequent, unstandardized laboratory tests.
*diagnosis*	**Care documentation**: Structured record of active problems.
*hospital*	**Administration**: Hospital level survey information: bed size, teaching status, and US region.
*infusionDrug*	**Care documentation**: Continuous infusions administered.
*intakeOutput*	**Care documentation**: Intake and output recorded for patients.
*lab*	**Care documentation**: Laboratory measurements for patient derived specimens.
*medication*	**Care documentation**: Prescribed medications usually interfaced from a local pharmacy system.
*microLab*	**Care documentation**: Manually entered microbiology information.
*note*	**Care documentation**: Semi-structured notes entered by the physician or physician extender responsible.
*nurseAssessment*	**Care documentation**: Documentation for patient items such as pain, psychosocial status, etc.
*nurseCare*	**Care documentation**: Documentation for patient items such as nutrition, wound care, drain/tube care, restraints, etc.
*nurseCharting*	**Care documentation**: Primary location for information charted at the bed side such as vital signs.
*pastHistory*	**Care documentation**: Structured list detailing patient's health status prior to presentation in the unit.
*patient*	**Administration**: Demographic and administrative information regarding the patient and their unit/hospital stay.
*physicalExam*	**Care documentation**: Semi-structured results of physical examinations performed.
*respiratoryCare*	**Care documentation**: Documentation for airway structure, cuff pressures, and other respiratory related details.
*respiratoryCharting*	**Care documentation**: Primary location for ventilator setting information including tidal volumes, pressure settings, etc.
*treatment*	**Care documentation**: Structured list detailing active treatments provided to the patient
*vitalAperiodic*	**Monitor data**: Unevenly sampled vital sign measurements such as non-invasive blood pressure.
*vitalPeriodic*	**Monitor data**: Five minute medians for continuous vital sign measurements such as invasive blood pressure.
Short descriptions of data contained in the table are provided. APACHE: Acute Physiology, Age, and Chronic Health Evaluation.	

**Table 5:** Hospital level information.

**Hospital level factor**	**Number of hospitals (%)**	**Number of patients (%)**
Bed capacity		
<100	46 (22.12%)	12,593 (6.27%)
100–249	62 (29.81%)	41,966 (20.89%)
250–499	35 (16.83%)	45,716 (22.76%)
>=500	23 (11.06%)	75,305 (37.49%)
Unknown	42 (20.19%)	25,279 (12.59%)
Teaching status		
False	189 (90.87%)	149,181 (74.27%)
True	19 (9.13%)	51,678 (25.73%)
Region		
Midwest	70 (33.65%)	65,950 (32.83%)
Northeast	13 (6.25%)	14,429 (7.18%)
South	56 (26.92%)	60,294 (30.02%)
West	43 (20.67%)	46,348 (23.07%)
Unknown	26 (12.50%)	13,838 (6.89%)
Information includes the region of the US the hospital is located in, whether it is a teaching hospital, the bed capacity, and the number of patients with data available for these hospital subtypes.		

**Table 6:** Organ system for problems documented during patient unit stays.

**Diagnosis group**	**Number of patients (%)**
Cardiovascular	104,264 (11.15%)
Pulmonary	64,222 (8.15%)
Neurologic	51,609 (7.28%)
Renal	43,009 (6.38%)
Endocrine	35,519 (6.15%)
Gastrointestinal	35,223 (6.10%)
Infectious diseases	20,316 (6.01%)
Hematology	19,611 (5.32%)
Burns/trauma	9,208 (5.13%)
Oncology	7,954 (4.72%)
Toxicology	7,185 (4.47%)
Surgery	5,723 (3.97%)
General	1,698 (3.91%)
Transplant	770 (3.75%)
Obstetrics/gynecology	46 (3.52%)
Genitourinary	26 (3.18%)
Musculoskeletal	19 (2.98%)
More than one problem can be documented for a single patient, and therefore the percentages will add up to greater than 100%.	

**Table 7:** Data available in *vitalPeriodic* table, including the number of patients who have at least one measurement, the total number of observations available, and the average number of observations available per patient for patients who have at least one measurement recorded.

**Data type**	**Column name**	**Number of patients (%)**	**Total number of observations (average patient-wise)**
Heart rate	heartrate	192277 (95.73%)	145,979,794 (759.2)
Peripheral oxygen saturation	sao2	189646 (94.42%)	132,908,266 (700.8)
Respiration rate	respiration	178051 (88.64%)	128,501,032 (721.7)
ST level	st2	98886 (49.23%)	59,949,273 (606.2)
ST level	st1	95643 (47.62%)	56,604,917 (591.8)
ST level	st3	92752 (46.18%)	55,201,239 (595.1)
Invasive mean blood pressure	systemicmean	46975 (23.39%)	28,060,870 (597.4)
Invasive systolic blood pressure	systemicsystolic	46667 (23.23%)	27,834,959 (596.5)
Invasive diastolic blood pressure	systemicdiastolic	46661 (23.23%)	27,833,847 (596.5)
Central venous pressure	cvp	28698 (14.29%)	19,157,758 (667.6)
Temperature	temperature	19419 (9.67%)	13,203,289 (679.9)
Mean pulmonary artery pressure	pamean	10893 (5.42%)	4,150,132 (381.0)
Diastolic pulmonary artery pressure	padiastolic	10792 (5.37%)	4,120,636 (381.8)
Systolic pulmonary artery pressure	pasystolic	10789 (5.37%)	4,121,138 (382.0)
End tidal carbon dioxide concentration	etco2	8346 (4.16%)	4,423,333 (530.0)
Intracranial pressure	icp	1634 (0.81%)	2,631,227 (1610.3)

**Table 8:** Data completion grouped by table and tabulated by hospitals.

**Table Name**	**Coverage**				
	**None**	**Low**	**Medium**	**High**	**Excellent**
*admissionDx*	0.48	0.48	5.77	15.38	77.88
*admissionDrug*	41.35	24.52	19.23	2.88	12.02
*allergy*	10.58	20.67	63.46	5.29	0.00
*apacheApsVar*	0.00	0.48	6.73	14.90	77.88
*apachePredVar*	0.00	0.48	6.73	14.90	77.88
*apachePatientResult*	8.65	0.96	16.83	12.98	60.58
*carePlanCareProvider*	0.96	0.96	12.02	12.98	73.08
*carePlanEOL*	53.85	46.15	0.00	0.00	0.00
*carePlanGeneral*	0.48	0.00	0.48	2.40	96.63
*carePlanGoal*	62.98	27.40	0.96	4.33	4.33
*carePlanInfectiousDisease*	53.85	38.94	6.73	0.48	0.00
*customLab*	92.79	4.81	0.48	0.48	1.44
*diagnosis*	0.48	0.48	11.54	11.54	75.96
*infusionDrug*	26.92	16.35	40.38	9.62	6.73
*intakeOutput*	2.40	3.85	5.29	12.02	76.44
*lab*	0.48	0.00	0.48	2.88	96.15
*medication*	16.35	7.21	2.40	1.92	72.12
*microLab*	89.42	5.77	3.85	0.96	0.00
*note*	0.00	0.00	3.37	16.83	79.81
*nurseAssessment*	92.31	1.92	0.96	0.00	4.81
*nurseCare*	93.27	0.96	0.96	0.00	4.81
*nurseCharting*	0.48	0.96	1.92	4.33	92.31
*pastHistory*	0.48	0.48	4.33	17.31	77.40
*physicalExam*	0.48	0.48	3.85	17.79	77.40
*respiratoryCare*	24.52	41.35	33.17	0.48	0.48
*respiratoryCharting*	11.06	15.38	35.58	9.62	28.37
*treatment*	6.25	3.37	12.98	11.54	65.87
*vitalAperiodic*	0.96	0.00	3.85	5.29	89.90
*vitalPeriodic*	0.96	0.00	3.37	2.40	93.27
Data completion is assessed by the percent of patient unit stays with data. For example, if between 20-60% of patientUnitStayId at a hospital have data, then we term this medium coverage, and 5.77% of hospitals have medium coverage for *admissionDx* . Coverage groups are: none (0%), low (0-20%), medium (20-60%), high (60-80%), and excellent (80-100%). Note that this table does not necessarily represent reliability of data collection as the expected prevalence of documentation for each table varies.					
